# Prognostic impacts of extracranial metastasis on non‐small cell lung cancer with brain metastasis: A retrospective study based on surveillance, epidemiology, and end results database

**DOI:** 10.1002/cam4.3562

**Published:** 2020-12-15

**Authors:** Miao Wang, Qiuji Wu, Jun Zhang, Guizhen Qin, Tian Yang, Yixin Liu, Xulong Wang, Boyu Zhang, Yongchang Wei

**Affiliations:** ^1^ Department of Radiation and Medical Oncology Zhongnan Hospital of Wuhan University Wuhan Hubei Province China; ^2^ Hubei Key Laboratory of Tumor Biological Behaviors Zhongnan Hospital of Wuhan University Wuhan Hubei Province China; ^3^ Hubei Cancer Clinical Study Center Zhongnan Hospital of Wuhan University Wuhan Hubei Province China

**Keywords:** brain metastasis, extracranial metastasis, NSCLC, prognosis

## Abstract

This study was designed to investigate the prognostic value of the number and sites of extracranial metastasis (ECM) in NSCLC patients with BM. NSCLC patients with BM from the Surveillance, Epidemiology, and End Results (SEER) database from 2010 to 2015 were enrolled in analysis. Patients from 2010 to 2013 were included in the training set and those from 2014 to 2015 in the validation set. ECM sites among different subtypes of NSCLC were compared by Chi‐square tests. Kaplan–Meier methods and Cox regression models were performed to analyze survival data. Competing‐risks analysis was used to predict cumulative incidence rates for CSS and non‐CSS cause. We included 5974 patients in the training cohort and 3561 patients in the validation cohort. Most (nearly 80%) NSCLC patients with BM showed 0–1 involved extracranial organ, with the most and least common ECM organ being bone and distant lymph nodes (DLNs) among all subtypes of NSCLC, respectively. The number of involved extracranial organs was an independent prognostic factor for patients with BM from NSCLC (*p* < 0.001). Patients with 0–1 ECM had better survival than those with larger number of involved extracranial organs (*p* < 0.001). Cumulative incidence rates for CSS were increased with the number of ECM raising (*p* < 0.001). All involved extracranial organs were associated with worse survival (*p* < 0.05). In patients with single‐organ ECM, we observed a better prognosis in lung and bone metastasis, while liver metastasis showed worst survival. But the difference in survival in these patient groups was relatively small. Patients with liver metastasis had higher cumulative incidence rates for CSS than that in patients with lung and bone metastasis (*p* < 0.05). More extracranial metastases were associated with poor prognosis in NSCLC patients with BM and ECM sites showed limited effect on survival. Tailored treatments would be reasonable for BM patients from NSCLC with different metastasis patterns.

## Graphical abstract

Prognostic value of extracranial organ involvement the Surveillance, Epidemiology and End Results (SEER) database Tailored treatments would be reasonable for BM patients from NSCLC with different metastasis patterns.

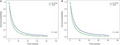

## 1 INTRODUCTION

Lung cancer is the most common malignancy and the leading cause of cancer‐related death worldwide.^1^ Approximately, 85% of lung cancer is NSCLC and up to 70% of NSCLC patients present with advanced disease.^2^, ^3^ 10%–20% of NSCLC patients have brain metastasis (BM) at their first diagnosis, and it is estimated that 25%–50% of NSCLC patients will ultimately develop BM during their disease course.^4^, ^5^ BM from NSCLC is associated with a dismal prognosis with 2‐year overall survival of no more than 10%–20% and imposes a distinct clinical challenge for clinicians.^6^, ^7^ BM from NSCLC is a highly complex and genetically heterogeneous disease. Considerable efforts have been taken to forecast the outcome of NSCLC patients with BM.

Various models have been proposed to prognosticate patients with BM from NSCLC. Commonly used models with recognized clinical pertinence include: (a) the recursive partitioning analysis (RPA) indices, an early prognostic index, which do not take into consideration the primary site as a prognostic factor;^8^ (b) to overcome the limitation of PRA, the disease‐specific graded prognostic assessment (DS‐GPA) tool was postulated, which stratified patients by primary tumor type and DS‐GPA tool for NSCLC patients with BM subdivide patients into one of four categories according to age at diagnosis, Karnofsky performance status (KPS), ECM, and number of BM;^9^ (c) the updated graded prognostic assessment for lung cancer that integrated prognosis‐associated molecular markers (Lung‐mol GPA).^10^ The latter incorporates a panel of prognostic factors associated with BM from NSCLC on the basis of DS‐GPA, including epidermal growth factor receptor (*EGFR*) and anaplastic lymphoma kinase (*ALK*) mutation status. According to Lung‐mol GPA, younger age, better performance status, absence of ECM, a smaller number of BM, and the *EGFR* mutation or *ALK* rearrangement predict a favorable survival. However, there is also evidence showing that the number of involved extracranial organs is related to the prognosis of cancer patients that develop BM. A study including 196 patients with BM from breast cancer elucidated that the number of involved extracranial organs is an independent prognostic factor, in addition to KPS and the number of BM.^11^ The role of the number of extracranial organs involvement in NSCLC with BM has not been clearly demonstrated. In addition, specific extracranial organ metastasis might lead to distinct prognosis. Adding the information of number of extracranial organs involvement and specific metastatic sites to conventional prognostic models might improve their prognosticating value.

Thus, in the present study, we assessed the prognostic role of the number of involved extracranial organs among NSCLC patients with BM based on the data from SEER database. We also intended to clarify the ECM profiles of NSCLC patients with BM and explore the prognostic characteristics among different ECM sites.

## 2 PATIENTS AND METHODS

### 2.1 Cohort population

This was a retrospective research employing information from the SEER database, a public available cancer statistics database, which is constitutive of 18 cancer registries in United States and covers about 28% of the total population of the United States (https://seer.cancer.gov/data/). Informed consent was waived for the use of public data from the SEER.

Patients with BM from NSCLC between 2010 and 2015 were included in this study following defined inclusion and exclusion criteria. The inclusion criteria were as follows: (a) age at diagnosis >=18 years old; (b) patients with confirmed pathological diagnosis (biopsy or surgical samples); (c) with confirmed BM from NSCLC. Exclusion criteria were: (a) patients with unknown metastasis information; (b) evidence of other coexisting malignancies.

### 2.2 Data extraction

Data regarding demographic features, TNM stages, pathology subtypes, involved extracranial organs and their numbers, anticancer treatments, and survival time were extracted from the database. Patients with BM from NSCLC between 2010 and 2013 were studied as the training cohort and those between 2014 and 2015 were explored as the validation cohort.

### 2.3 Statistical analysis

Descriptive statistics were performed to summarize patients’ demographic and clinical characteristics among patients with an involvement of 0, 1, 2, ≥3 extracranial organs. Categorized data were analyzed by Chi‐square tests. The Kaplan–Meier analyses were used to generate the survival curves and the Log‐Rank test was employed to compare the difference among the curves. Univariate and multivariate Cox regression models were utilized to identify prognostic risk factors of overall survival (OS) and lung cancer‐specific survival (CSS). The Fine and Gray's proportional subdistribution hazard model was fitted to predict cumulative incidence function (CIF) of mortality from CSS and other cause in a competing‐risks setting and the Gray test was applied to compare cumulative incidence differences among groups. OS was defined as the time from diagnosis to death from any cause and CSS was defined as the time from diagnosis to death from lung cancer. A *p* value <0.05 was considered statistically significant and all statistical tests were two‐sided. All statistical analyses were conducted using GraphPad Prism 7 (GraphPad Software) and SPSS statistics version 23.0 (IBM SPSS Statistics).

## 3 RESULTS

### 3.1 Patient aharacteristics

A total of 5974 NSCLC patients with BM diagnosed from 2010 to 2013 were enrolled in the training cohort and patient clinical characteristics are summarized in Table 1, among whom 2750 (46.03%) patients had zero involved extracranial organ, 1933 (32.36%) patients had one involved extracranial organ, 970 (16.24%) patients had two involved extracranial organs, and 321(5.37%) had three or more involved extracranial organs. Among 3561 patients in validation cohort from 2014 to 2015, there were 1588 (44.59%), 1107 (31.09%), 647 (18.17%), and 219 (6.14%) cases presenting with 0‐, 1‐, 2‐, ≥3‐site ECM, respectively. (Table S1).

**TABLE 1:** Characteristics of NSCLC patients with brain metastasis

Characteristics	Total	The number of involved extracranial organs				*p* value
		0	1	2	≥3	
	5974 (100%)	2750 (46.03%)	1933 (32.36%)	970 (16.24%)	321 (5.37%)	
**Histopathology**						**<0.001**
Adenocarcinoma	4519 (75.64%)	1997 (72.62%)	1500 (77.60%)	767 (79.07%)	255 (79.44%)	
Squamous cell carcinoma	898 (15.03%)	471 (17.13%)	161 (13.50%)	127 (13.09%)	39 (12.15%)	
Large cell carcinoma	209 (3.50%)	119 (4.33%)	61 (3.16%)	20 (2.06%)	9 (2.80%)	
Other	348 (5.83%)	163 (5.93%)	111 (5.74%)	56 (5.77%)	18 (5.61%)	
**Age**						0.288
21–49	541 (9.06%)	245 (8.87%)	162 (8.38%)	99 (10.21%)	36 (11.21%)	
50–59	1675 (28.04%)	1675 (0.27%)	550 (28.45%)	278 (28.66%)	97 (30.22%)	
≥60	3758 (62.91%)	3758 (63.85%)	1221 (63.17%)	593 (61.13%)	188 (58.57%)	
**Race**						**<0.001**
White	4619 (77.32%)	2168 (78.84%)	1484 (76.77%)	732 (75.46%)	235 (73.21%)	
Black	771 (12.91%)	371 (13.49%)	252 (13.04%)	112 (11.55%)	36 (11.21%)	
Other^a^	584 (9.78%)	211 (7.67%)	197 (10.19%)	126 (12.99%)	50 (15.58%)	
**Gender**						0.685
Male	2910 (48.71%)	132 (48.33%)	937 (48.47%)	478 (49.28%)	166 (51.71%)	
Female	3064 (51.29%)	142 (51.67%)	996 (51.53%)	492 (50.72%)	155 (48.29%)	
**Marital status**						**0.01**
Married	2572 (43.05%)	1261 (45.85%)	790 (40.87%)	389 (40.10%)	132 (41.12%)	
Unmarried	3179 (53.21%)	139 (50.62%)	1066 (55.15%)	545 (56.19%)	176 (54.83%)	
Unknown	223 (3.73%)	97 (3.53%)	77 (3.98%)	36 (3.71%)	13 (4.05%)	
**Differentiation**						0.259
Well	139 (2.33%)	56 (2.04%)	50 (2.59%)	25 (2.58%)	8 (2.49%)	
Moderately	796 (13.32%)	382 (13.89%)	264 (13.66%)	117 (12.06%)	33 (10.28%)	
Poorly	1760 (29.46%)	907 (32.98%)	547 (28.30%)	241 (24.85%)	65 (20.25%)	
Undifferentiated	86 (1.44%)	50 (1.82%)	23 (1.19%)	10 (1.03%)	3 (0.93%)	
Unknown	3193 (53.45%)	1355 (49.27%)	1049 (54.27%)	577 (9.48%)	212 (66.04%)	
**T Stage**						**<0.001**
T1	743 (12.44%)	455 (16.55%)	207 (10.71%)	75 (7.73%)	6 (1.87%)	
T2	1765 (29.54%)	1023 (37.20%)	524 (27.11%)	184 (18.97%)	34 (10.59%)	
T3	1541 (25.80%)	628 (22.84%)	530 (27.42%)	290 (29.90%)	93 (28.97%)	
T4	1925 (32.22%)	644 (23.42%)	672 (34.76%)	421 (43.40%)	188 (58.57%)	
**N Stage**						**<0.001**
N0	1301 (21.78%)	775 (28.18%)	355 (18.37%)	140 (14.43%)	31 (9.66%)	
N1	520 (8.70%)	276 (10.04%)	154 (7.97%)	70 (7.22%)	20 (6.23%)	
N2	2829 (47.36%)	127 (46.18%)	942 (48.73%)	470 (48.45%)	147 (45.79%)	
N3	1183 (19.80%)	372 (13.53%)	434 (22.45%)	263 (27.11%)	114 (35.51%)	
Unknown	141 (2.36%)	57 (2.07%)	48 (2.48%)	27 (2.78%)	9 (2.80%)	
**Surgery**						**<0.001**
Yes	135 (1.96%)	86 (3.13%)	24 (1.24%)	5 (0.52%)	2 (0.62%)	
No	5839 (98.04%)	2664 (96.87%)	1909 (98.76%)	965 (99.48%)	319 (99.38%)	
**Radiation therapy**						0.609
Yes	5857 (97.74%)	2689 (97.78%)	1886 (97.57%)	947 (97.63%)	317 (98.75%)	
No	117 (2.26%)	61 (2.22%)	47 (2.43%)	23 (2.37%)	4 (1.25%)	
**Chemotherapy**						0.768
Yes	3749 (62.76%)	1707 (62.07%)	1229 (63.58%)	611 (62.99%)	202 (62.93%)	
No	2225 (37.24%)	1043 (37.93%)	704 (36.42%)	359 (37.01%)	119 (37.07%)	

Abbreviations: NSCLC, non‐small cell lung cancer.

^a^ American Indian/AK Native, Asian/Pacific Islander (in bold).

### 3.2 The association between clinicopathological characteristics and number of ECM sites

Clinicopathological characteristics including histopathology, race, marital status, N stage, and T stage showed statistically significant differences among patients with 0‐, 1‐, 2‐, ≥3‐organ ECM. It is worth noting that patients with larger number of involved extracranial organs were more likely to have a higher rate of T4 stage (23.42% vs. 34.76% vs. 43.40% vs. 58.57%, *p* < 0.001), as well as N3 stage (13.53% vs. 22.45% vs. 27.11% vs. 35.51%, *p* < 0.001). Other factors did not show obvious association with number of ECM sites. As for therapy, patients with smaller number of involved extracranial organs received more surgery (for primary site), but in all the rate of surgery was low, with only 1.96% of all patients receiving surgery. There were no differences in different number of ECM among patients receiving chemotherapy or radiotherapy. And 37.24% of patients with BM from NSCLC did not undergo chemotherapy in the training set. In validation cohort, the possibility of T4 stage or N3 stage increased with the number of involved extracranial organs increasing (T4: 22.67% vs. 35.32% vs. 42.66% vs. 56.62%, *p* < 0.001; N3: 16.44% vs. 26.11% vs. 29.98% vs. 38.36%, *p* < 0.001). There are 32.88% of NSCLC patients with BM did not receive systemic chemotherapy in validation cohort.

### 3.3 ECM Sites

In the training cohort, the most and least frequent ECM site were bone (65.57% [2114/3224]) and distant lymph nodes (dLNs) (5.24% [169/3224]), respectively. This was similar in the validation cohort, with the most and least frequent involved extracranial organs being bone and dLNs and the prevalence being 70.24% (1386/1973) and 5.01% (99/1973), respectively.

**FIGURE 1:**
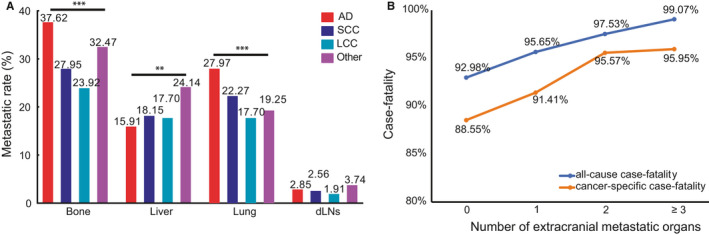
(A) Distribution of extracranial metastatic organs based on the pathology subtype of NSCLC and (B) all‐cause case‐fatality and cancer‐specific case‐fatality of different number of extracranial metastatic organs. Abbreviation: AD: adenocarcinoma; SCC: squamous cell carcinoma; LCC: large cell carcinoma, dLNs: distant lymph nodes, NSCLC: non‐small cell lung cancer. (*p < 0.05, **p < 0.01, ***p < 0.001)

We queried if different histopathological subtypes showed varied ECM patterns and we explored ECM sites and their prevalence in adenocarcinoma (AD), squamous cell carcinoma (SCC), large cell carcinoma (LCC), and other rare subtypes. Interestingly, we found that in the training group, bone and dNLs were still the most and the least common ECM sites among all subtypes of NSCLC (Figure 1A). We noted considerable variance in ECM patterns among different subtypes of NSCLC in the training cohort. AD patients had the highest rate of extracranial bone (37.62%) and lung (27.97%) metastasis and LCC patients showed the lowest rate of extracranial bone (23.92%) and lung (17.70%) metastasis. Besides, extracranial liver metastasis was seen highest in other rare subtype and lowest in AD patients with BM. There was no statistical difference in extracranial dLNs metastasis. The validation cohort showed similar ECM sites distribution (Figure S1A).

### 3.4 The prognostic value of number of ECM

**FIGURE 2:**
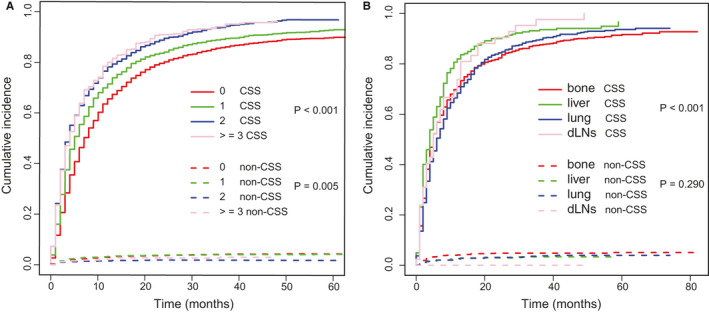
Cumulative incidence function curves of CSS and non‐CSS cause according to (A) the number and (B) sites of ECM. Abbreviation: CSS: cancer‐specific survival; ECM: extracranial metastasis

**FIGURE 3:**
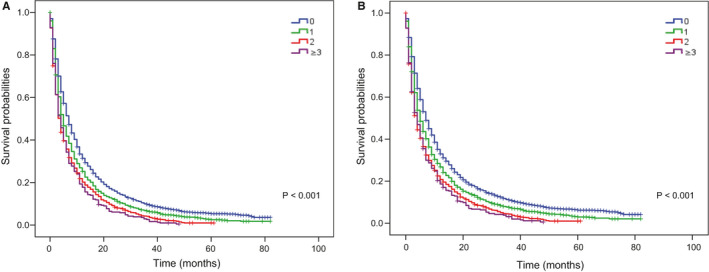
Kaplan–Meier curves of (A) OS and (B) CSS by the number of extracranial metastatic organs group in patients with BM from NSCLC. Abbreviation: OS: overall survival; CSS: cancer‐specific survival; BM: brain metastasis, NSCLC: non‐small cell lung cancer

**FIGURE 4:**
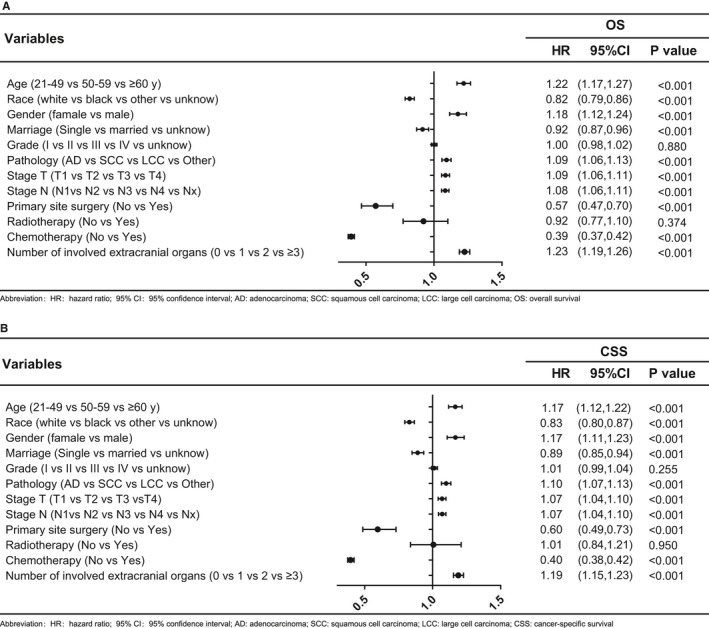
Multivariable Cox regression analyses and forest plots of prognostic factors of (A) OS and (B) CSS in NSCLC patients with BM. Abbreviation: OS: overall survival; CSS: cancer‐specific survival; NSCLC: non‐small cell lung cancer; BM: brain metastasis, HR: hazard ratio; 95% CI: 95% confidence interval, AD: adenocarcinoma; SCC: squamous cell carcinoma; LCC: large cell carcinoma

After a median follow‐up of 52 months, 5670 all‐cause deaths and 5437 lung cancer‐related deaths occurred in 5974 patients in the training cohort, resulting in an extremely high all‐cause case‐fatality of 94.91% (5670/5974) and cancer‐specific case‐fatality of 91.01% (5437/5974). We found that both all‐cause case‐fatality and cancer‐specific case‐fatality increased with the number of ECM elevating (all‐cause case‐fatality: 92.98% [0 ECM] vs. 95.65% [1 ECM] vs. 97.53% [2 ECM] vs. 99.07% [≥3 ECM], *p* < 0.001, cancer‐specific case‐fatality: 88.55% [0 ECM] vs. 91.41% [1 ECM] vs. 95.57% [2 ECM] vs. 95.95% [≥3 ECM], *p* < 0.001). (Figure 1B). And cumulative incidence function (CIF) curves for CSS and other cause showed cumulative incidence rates for CSS surpassed that for other cause in patients with 0, 1, 2, ≥3 involved extracranial organs and cumulative incidence rates for CSS were increased gradually with time since initial diagnosis. Besides, we found that patients with larger number of ECM (≥1) showed higher cumulative incidence rates of CSS (*p* < 0.001) (Figure 2A). We confirmed these observations in the validation cohort (Figure S2A). Besides, the 6‐month and 1‐year overall survival (OS) and cancer‐specific survival (CSS) were also correlated with numbers of ECM organs (6‐month OS: 51.4% vs. 41.8% vs. 35.7% vs. 34.3%, *p* < 0.001; 6‐month CSS: 47.8% vs. 39.4% vs. 34.3% vs. 33.4%, *p* < 0.001; 1‐year OS: 31.4% vs. 25.2% vs. 20.2% vs. 17.8%, *p* < 0.001; and 1‐year CSS: 26.3% vs. 21.9% vs. 18.4% vs. 16.6%, *p* < 0.001; for 0‐, 1‐, 2‐, and ≥3‐ECM, respectively) (Table 2 and Table S2). The median OS of patients with 0, 1, 2, or ≥3 ECM were 7, 5, 4, and 4 months, respectively, (Figure 3A) and the median CSS was 7, 5, 4, and 4 months, respectively, (*p* < 0.001) in the training cohort (Figure 3B). Comparison of OS and CSS among different numbers of ECM organs in validation cohort is showed in Figure S3 (*p* < 0.001). And the number of involved extracranial sites showed a statistically significant effect on survival (OS: HR=1.23 [95% CI: 1.19–1.26], *p* < 0.001; CSS: HR=1.19 [95% CI: 1.15–1.23], *p* < 0.001) after adjustment for age, race, gender, histology, marital status, grade, and stage N and T, and treatment in training cohort (Figure 4). In the validation cohort, the number of involved extracranial organs was also confirmed as an independent prognostic indicator for survival (OS: HR = 1.16 [95% CI: 1.11–1.21], *p* < 0.001; CSS: HR = 1.14 [95% CI: 1.10–1.19], *p* < 0.001) (Figure S4). As for therapies, patients accepted cancer treatment of primary site surgery and chemotherapy had better survival than those who did not.

**TABLE 2:** Survival analysis among different numbers of involved extracranial organs

Survival rate	The number of involved extracranial organs				*p* value
	0	1	2	≥3	
6‐month OS	51.4%	41.8%	35.7%	34.3%	<0.001
1‐year OS	31.4%	25.2%	20.2%	17.8%	<0.001
6‐month CSS	47.8%	39.4%	34.3%	33.4%	<0.001
1‐year CSS	26.3%	21.9%	18.4%	16.6%	<0.001

### 3.5 Prognostic differences between different sites of ECM

**FIGURE 5:**
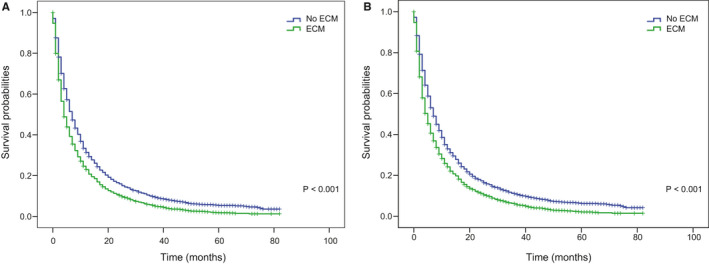
Comparison of (A) OS and (B) CSS among patients with or without ECM. Abbreviation: OS: overall survival; CSS: cancer‐specific survival; ECM: extracranial metastasis

**FIGURE 6:**
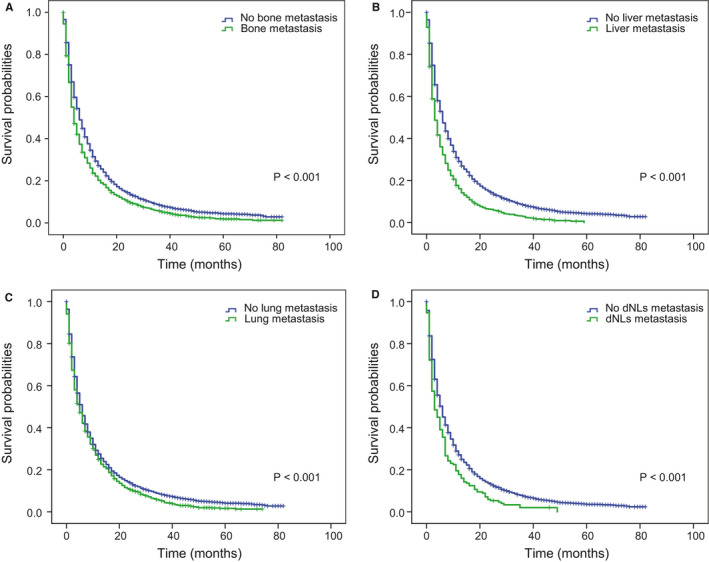
Kaplan–Meier curves of OS in patients according to extracranial metastasis sites. A, Extracranial bone metastasis; B, extracranial liver metastasis; C, extracranial lung metastasis; D, extracranial dLNs metastasis. Abbreviation: OS: overall survival; dLNs: distant lymph nodes.

Patients without ECM sites showed the better OS and CSS than these patients with ECM (Figure 5A,B and Figure S5A,B). And univariate cox analysis showed that extracranial metastatic organs in four organs were closely related to worse OS (bone: HR: 1.25, 95% CI [1.19–1.32], *p* < 0.001; liver: HR: 1.51, 95% CI: [1.41–1.61], *p* < 0.001; lung: HR: 1.14, 95% CI: [1.08–1.21], *p* < 0.001; dLNs: HR: 1.39, 95% CI [1.19–1.62], *p* < 0.001), as well as CSS (bone: HR: 1.18, 95% CI [1.12–1.25], *p* < 0.001; liver: HR: 1.38, 95% CI [1.29–1.49], *p* < 0.001; lung: HR: 1.07, 95% CI [1.01–1.15], *p* = 0.025; dLNs: HR: 1.26, 95% CI [1.08–1.47], *p* = 0.003) (Table 3) And similar results were found in the validation cohort (Table S2). And Kaplan–Meier curves were used to describe the survival data of OS more intuitively in both cohorts (Figure 6A–D and Figure S6A–D).

**TABLE 3:** Univariate analyses of OS and CSS in diverse extracranial metastatic organs

Variable	OS		CSS	
	HR (95% CI)	*p* value	HR (95% CI)	*p* value
Bone (metastasis vs. no metastasis)	1.25 (1.19–1.32)	<0.001	1.18(1.12–1.25)	<0.001
Liver (metastasis vs. no metastasis)	1.51(1.41–1.61)	<0.001	1.38(1.29–1.49)	<0.001
Lung (metastasis vs. no metastasis)	1.14(1.08–1.21)	<0.001	1.07(1.01–1.15)	0.025
dLNs (metastasis vs. no metastasis)	1.39 (1.19–1.62)	<0.001	1.26(1.08–1.47)	0.003

Abbreviations: 95% CI, 95% confidence interval; CSS, cancer‐specific survival; dLNs, distant lymph nodes; HR, hazard ratio; OS, overall survival.

**FIGURE 7:**
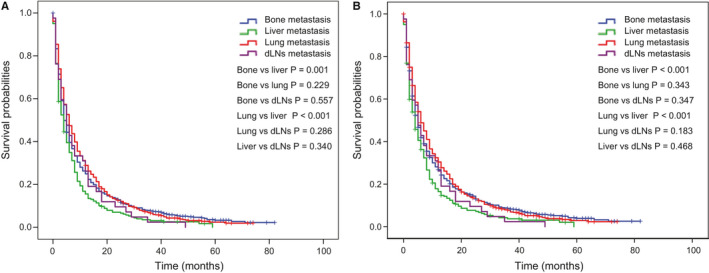
Comparison of (A) OS and (B) CSS of different extracranial metastasis status among patients with only one extracranial metastatic organ. Abbreviation: OS: overall survival; CSS: cancer‐specific survival

To further understand whether different ECM sites might impact on patients’ survival, we compared OS and CSS in patients with bong, lung, dLNs, and liver as the only ECM organ. We observed better prognosis of bone and lung metastasis, while liver metastasis showed worst survival in two cohort (Figure 7A,B and Figure S7A,B). However, the overall difference in survival in these patient groups was relatively small. And as the CIF curves (Figure 2B and Figure S2B) showed that patients with liver metastasis had a higher cumulative incidence rates while patients with bone and lung metastasis showed a lower cumulative incidence rates(*p* < 0.001).

## 4 DISCUSSION

BM from NSCLC remained a substantial contributor to high cancer mortality in advanced NSCLC patients despite the emergence of multimodal therapies that include a combination of surgery, radiotherapy, chemotherapy, immunotherapy, and targeted therapies.^12^, ^13^ And our results also revealed that NSCLC patients with BM had a high cancer mortality. Various factors affecting prognosis have been identified in previous studies, including age at diagnosis, KPS, ECM, number of BM, *EGFR* mutation, or *ALK* rearrangement.^8^, ^9^, ^10^ However, the way ECM impacted on patients’ prognosis has not been investigated clearly. In this study, by using large cohort of patients from the SEER database, we found that the number of ECM was significant prognostic factor of NSCLC patients with BM and the sites of ECM showed limited impact on survival.

In breast cancer, it was shown that the number of involved extracranial organs showed a significant impact on survival in patients with BM.^11^ A study involving 472 NSCLC patients with BM suggested that the number of involved extracranial organs was an additional important prognostic indictor apart from age at diagnosis, KPS, and the number of BM.^14^ The present cohort study, based on data of 9535 cases with NSCLC and BM from SEER database, who were divided into the training and validation cohort, elaborated the impact of the number of ECM on NSCLC cancer survival. Specifically, patients with zero involved extracranial organs had the best survival while 1, 2, ≥3 involved extracranial organs were related to worse survival (*p* < 0.001). The survival rates at 6‐months and 12‐months were decreased as the number of extracranial organs increased (*p* < 0.001). Cancer mortality were elevated with the number of extracranial organs increasing (*p* < 0.001), which may be attributable to lower willingness of patients and doctors to pursue aggressive therapy for patients with multiple metastasis and limited effectiveness of systemic chemotherapy for BM. In our study, there were nearly 40% patients with BM from NSCLC who did not receive systemic chemotherapy. On the contrary, we found that receiving salvage anticancer treatment such as surgery and chemotherapy were associated with favorable survival even in advanced diseases, suggesting that an appropriate antitumor treatment could bring survival benefits and possibly improve patients’ quality of life. The existing literatures has showed that primary site surgery was a favorable prognostic factor in oligometastatic stage IV NSCLC patients.^15^, ^16^ And in our study most (nearly 80%) patients had 0–1 ECM site. These could be explained why primary site surgery is an independent prognosis factor in our study.

Moreover, nowadays, there are advanced novel immunotherapies and targeted therapies that was reported to show increased blood–brain barrier penetration and realize high response rates and more durable control of BM, and advances in radiotherapies and minimally invasive surgical techniques, which are thought to improve the survival outcome of patients with BM.^7^, ^17^ For example, EGFR/ALK TKIs had significant intracranial activity and could achieve longer intracranial progression free survival, higher overall response rate than chemotherapy or radiotherapy, which have been proved in many retrospective and prospective studies.^18^, ^19^, ^20^, ^21^, ^22^ And some studies have confirmed that stereotactic radiotherapy (SRS) was a well‐tolerated safe option for patients with four or more BM.^23^, ^24^, ^25^ Besides, we also found that most patients with BM from NSCLC present with 0–1 ECM site and these patients showed superior survival. Therefore, selecting the optimum treatment for an individual patient and receiving aggressive treatment were mandatory in order to decrease the mortality and improve survival in the era of multimodal treatment.

In present study, bone was the most common ECM site and dLNs was the least common ECM site among different subtype, which is in agreement with previous studies. A retrospective study of 729 NSCLC patients reported that the most and least frequent metastasis site were bone and dLNs, respectively.^26^ Similarly, a previous study based on SEER database demonstrated that more than half of patients showed bone metastasis within metastatic NSCLC patients.^27^ Remarkably, we found that different subtypes showed difference among diverse ECM sites, which is probably because different subtypes have different genetic variation propensities, such as EGFR, ALK, and MET were seen more mutated in AD, while FGFR1 and FGFR3 mutation were more frequent in SCC.^28^, ^29^, ^30^, ^31^, ^32^ Indeed, different mutational profiles being prone to distinctive metastatic patterns in lung cancer have been elucidated. For example, EGFR was associated with bone and brain metastasis in AD patients.^33^, ^34^, ^35^ However, the exact molecular mechanism dictating the metastasis pattern of different histopathological subtypes on NSCLC remains unclear.

Besides, different extracranial sites involvement might lead to different survival outcomes. Interestingly, we found all different extracranial organs metastasis were associated with unfavorable OS and CSS in univariate analysis. And Kaplan–Meier curves also intuitively display this phenomenon. We investigate prognostic difference of ECM sites among patients with only one ECM site and found that there was small difference in survival in these patient groups. Well, a previous study suggested that the involved extracranial organs did not showed significant association with survival in NSCLC patients with only one or two ECM sites.^14^ These results hinted that the sites of ECM displayed limited impact on survival in patients with BM from NSCLC.

The retrospective research had several potential limitations. First, no information was provided in the SEER database regarding the number, size, location and presence of symptom of BM, and systemic treatment such as chemotherapy, targeted therapy, which lead to some significant prognostic information neglected. Second, metastatic information from SEER database was only available for bone, brain, liver, lung and distant lymph glands, and other distal sites such as adrenal glands are unclear, which may cause some metastasis sites ignored. Despite these limitations, we collected 9535 cases from SEER database, which offered a relatively large sample size to perform accurate and multiple forms of analysis. Moreover, based on dividing enrolled patients into training cohort and validation cohort according to year of diagnosis, we first identified that the number and site of extracranial metastases as impacting factors for survival in training set and we then further demonstrated their significance in a validation cohort, which suggested that our results and conclusion were convincing and credible.

## 5 CONCLUSION

Our research indicated that most (nearly 80%) NSCLC patients with BM had 0–1 extracranial organ involvement and the number of involved extracranial organs was an independent prognosis factor in NSLC patients with BM. Patients with 0–1 involved extracranial organs exhibited better survival compared patients with two or more ECM sites. And patients with larger number of ECM showed higher cumulative incidence rates of CSS (*p* < 0.001). And we illustrated the ECM sites among different subtypes of NSCLC and prognostic impact of different extracranial organs involvement. Bone and dLNs were the most and least common ECM organ for all subtypes of NSCLC and all involved extracranial organs were related to worse survival in univariate analysis. Besides, ECM sites showed limited impact on survival in patients with only one ECM site. Therefore, our findings hopefully provide more information for future study design and clinical decision.

## CONFLICT OF INTERESTS

The authors declare no competing interests.

## AUTHOR CONTRIBUTIONS

All authors were integral for the study and contributed to the research as noted below. Wang Miao: Data collection and data analysis, and writing of the manuscript; Wu Qiuji: Design and data analysis, and writing of the manuscript; Zhang Jun: Data analysis and data collection; Qin Guizhen: data analysis and writing of the manuscript; Liu Yixin and Wang Xulong: Data collection and data analysis; Zhang Boyu: Writing of the manuscript; Wei Yongchang: Design, administrative support, resources, funding support, and critical revision of the manuscript.

## Supporting information

Fig S1

Fig S2

Fig S3

Fig S4

Fig S5

Fig S6

Fig S7

Table S1

Table S2

Table S3

## Data Availability

The data in the current study are available and public in the Surveillance, Epidemiology, and End Results (SEER) database (https://seer.cancer. gov/data/).
