# Supplementary figures and images for: Prognostic impacts of extracranial metastasis on non‐small cell lung cancer with brain metastasis: A retrospective study based on surveillance, epidemiology, and end results database
Source: Cancer Med. 2020 Dec 15;10(2):471–82. doi: 10.1002/cam4.3562 (PMC7877345; doi:10.1002/cam4.3562)

A

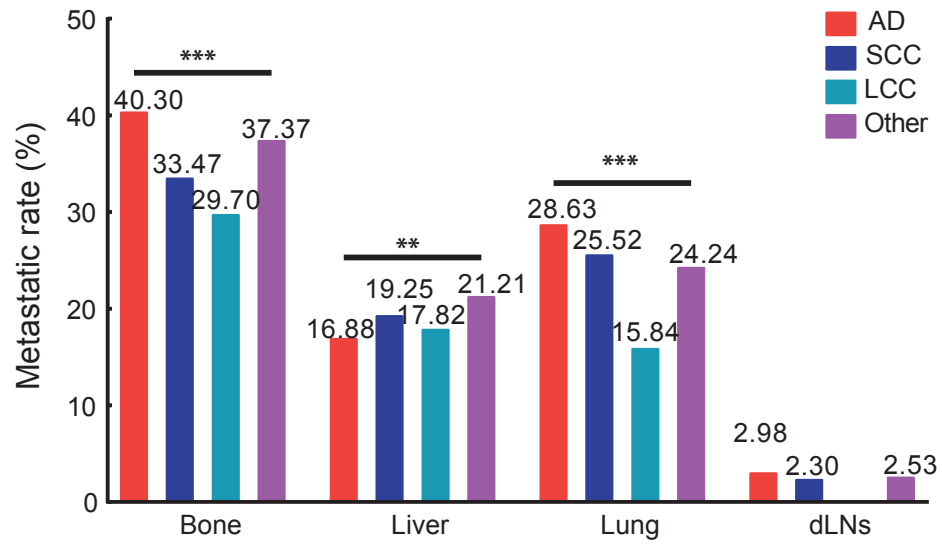

B

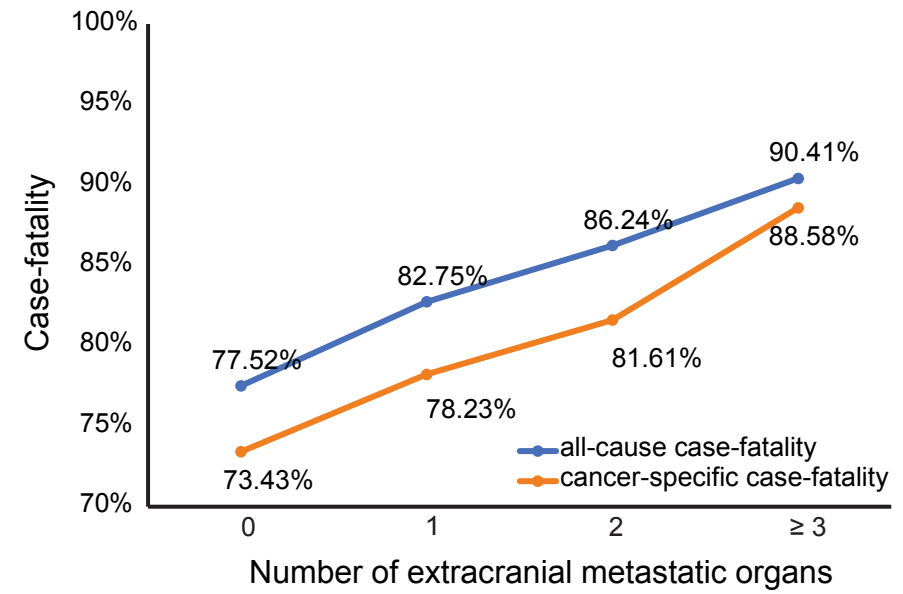

Supplementary figure 1

Supplement: Supplementary file 1 — Fig S1 [file CAM4-10-471-s001.pdf]

A

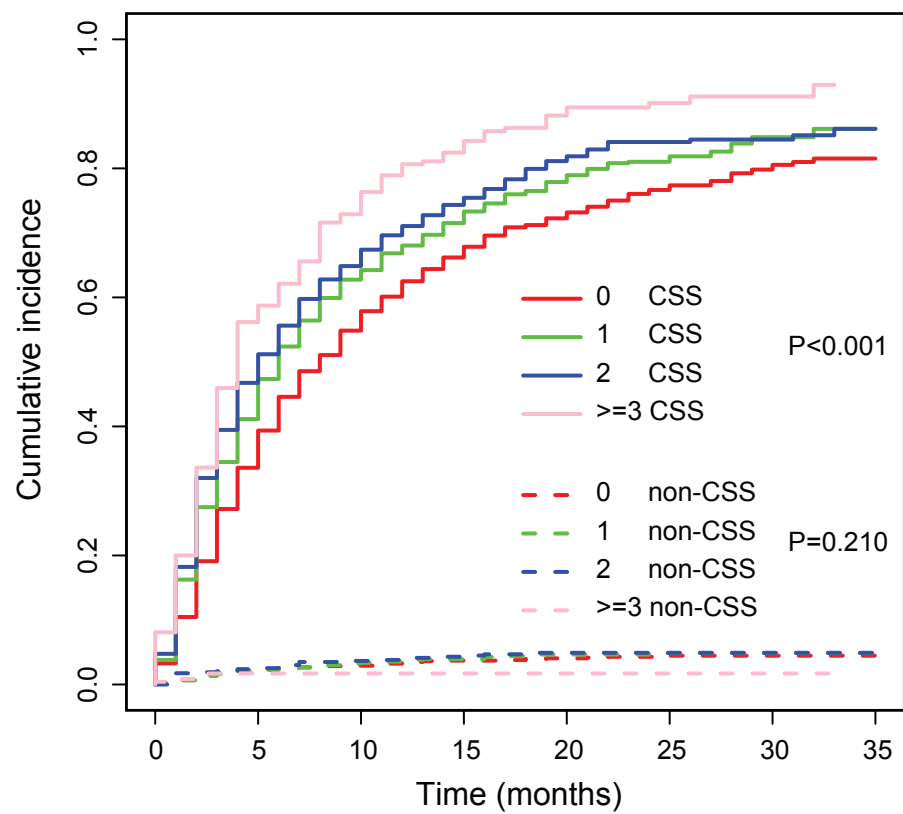

B

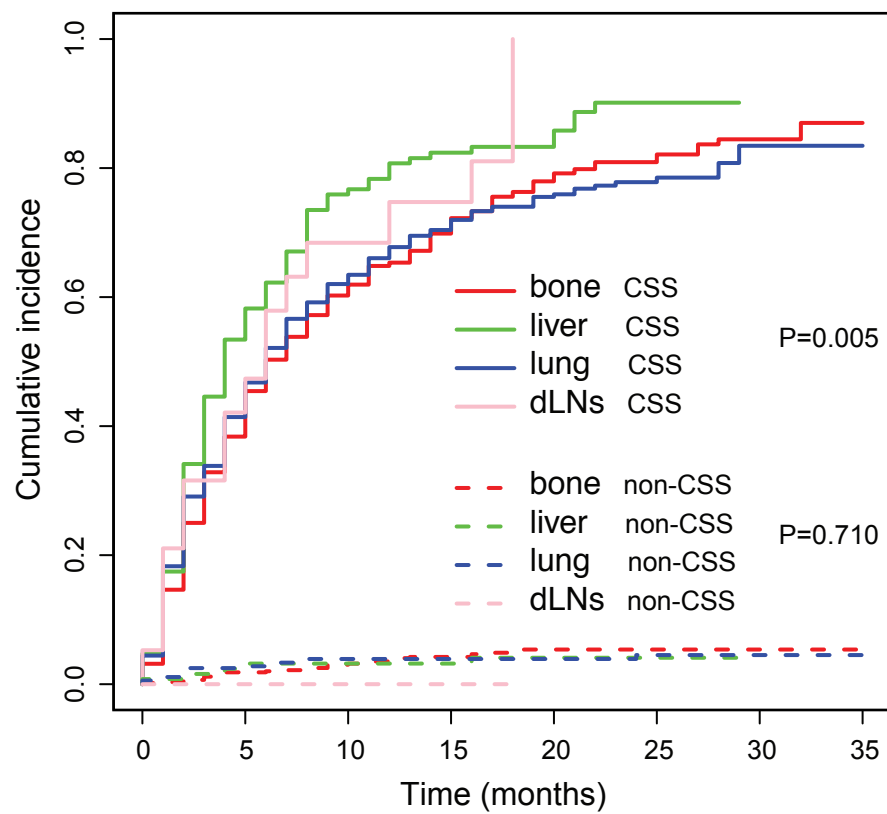

Figure 2

Supplement: Supplementary file 2 — Fig S2 [file CAM4-10-471-s002.pdf]

A

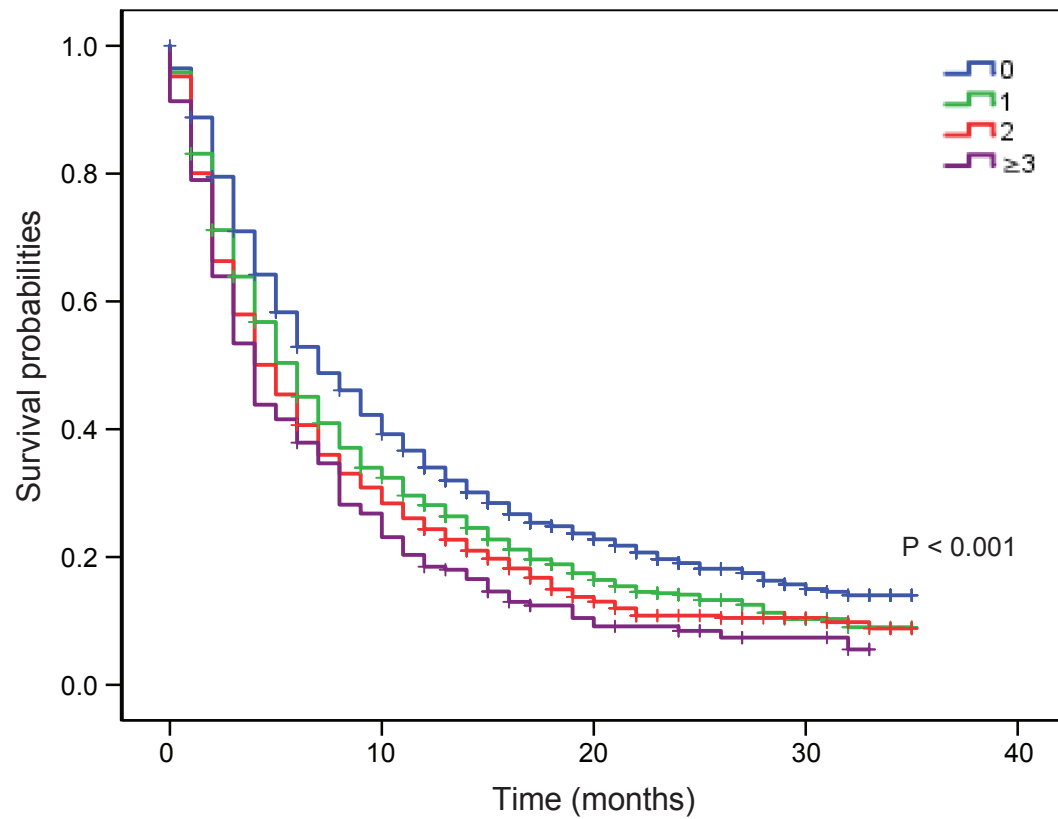

B

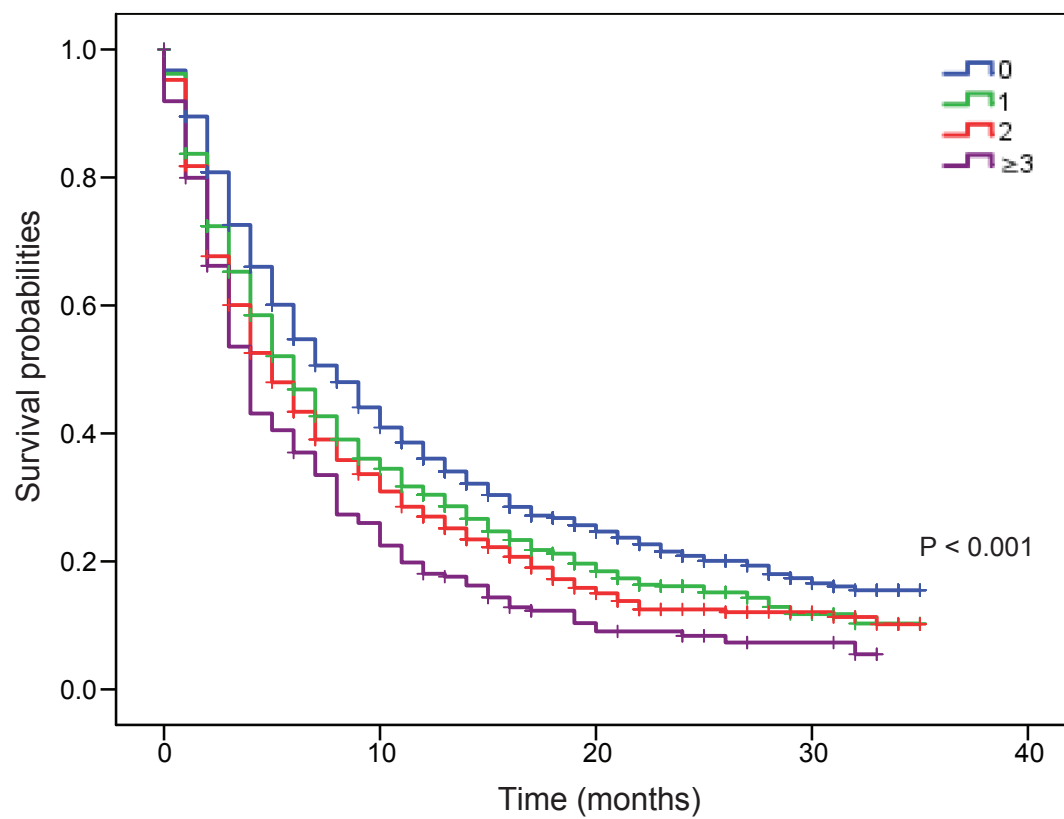

Supplementary figure 3

Supplement: Supplementary file 3 — Fig S3 [file CAM4-10-471-s003.pdf]

**A**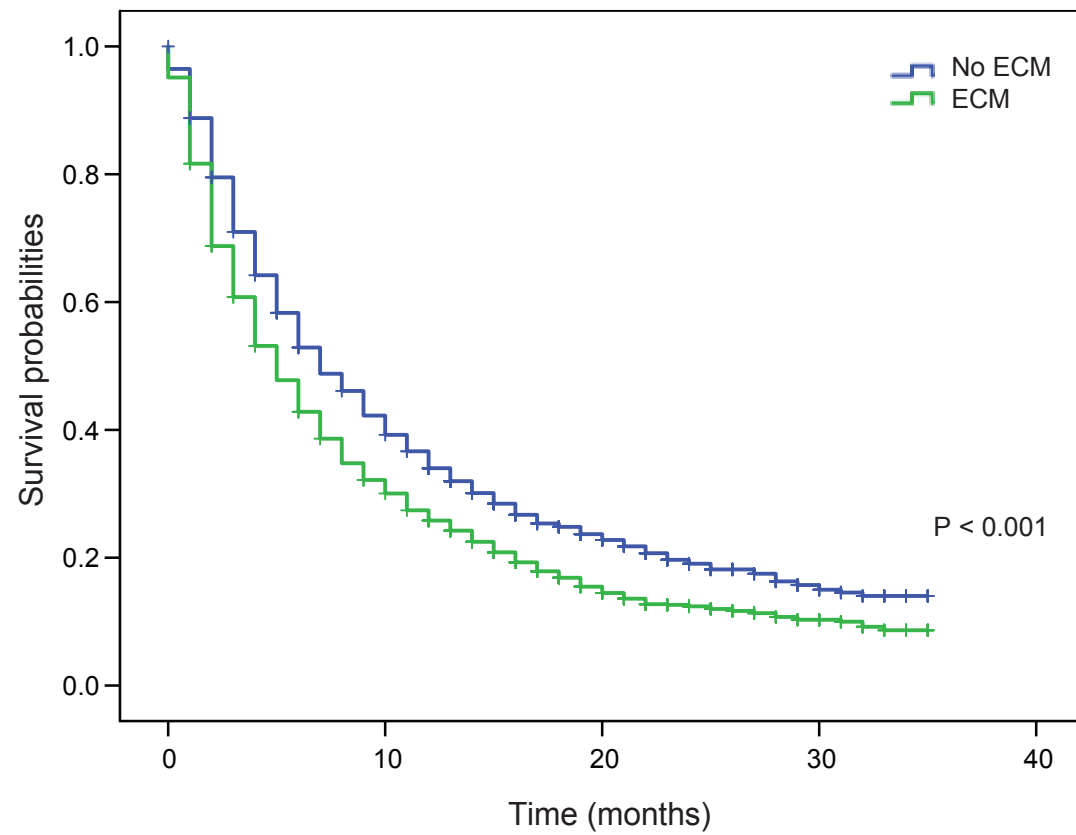**B**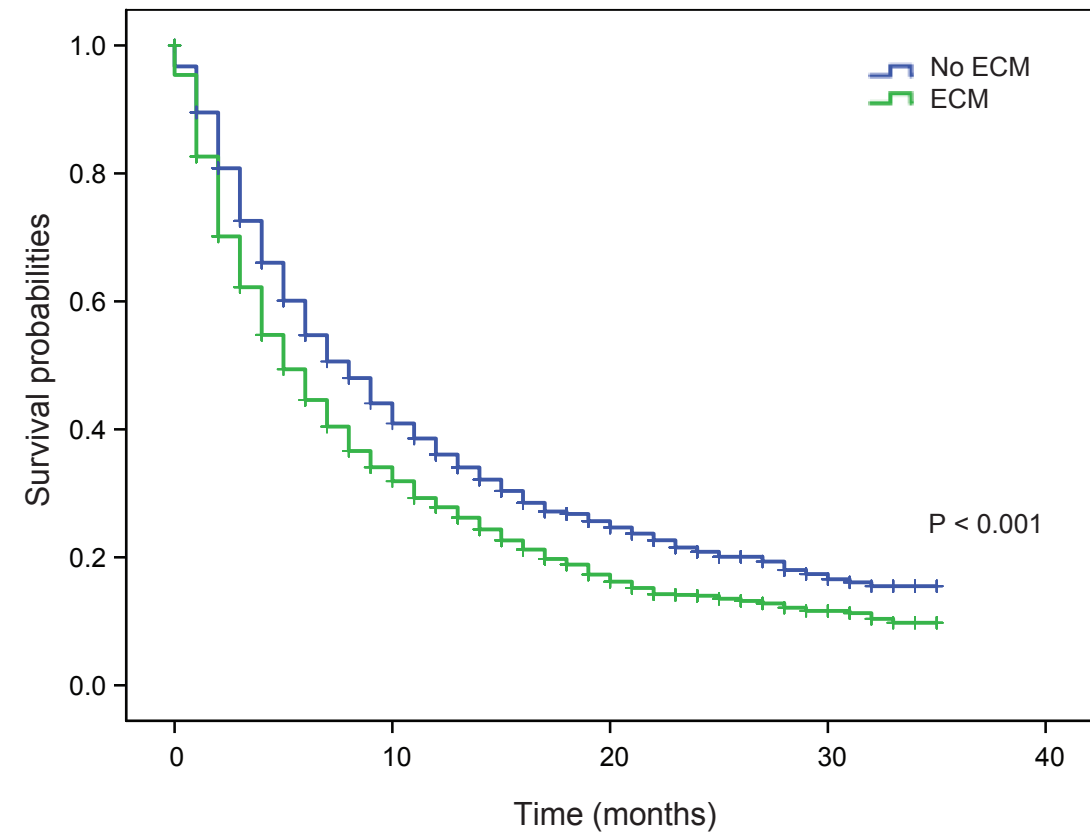

Supplementary figure 5

Supplement: Supplementary file 5 — Fig S5 [file CAM4-10-471-s005.pdf]

A

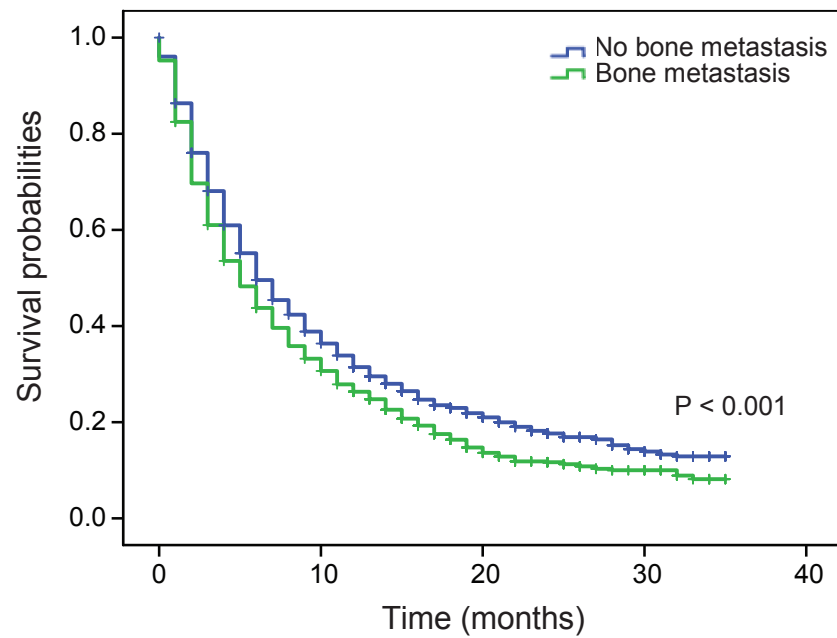

B

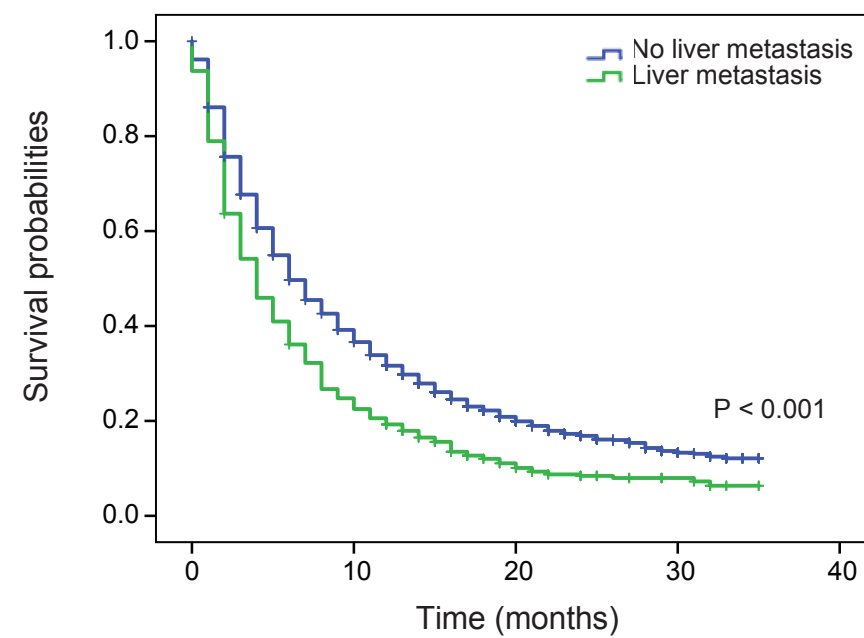

C

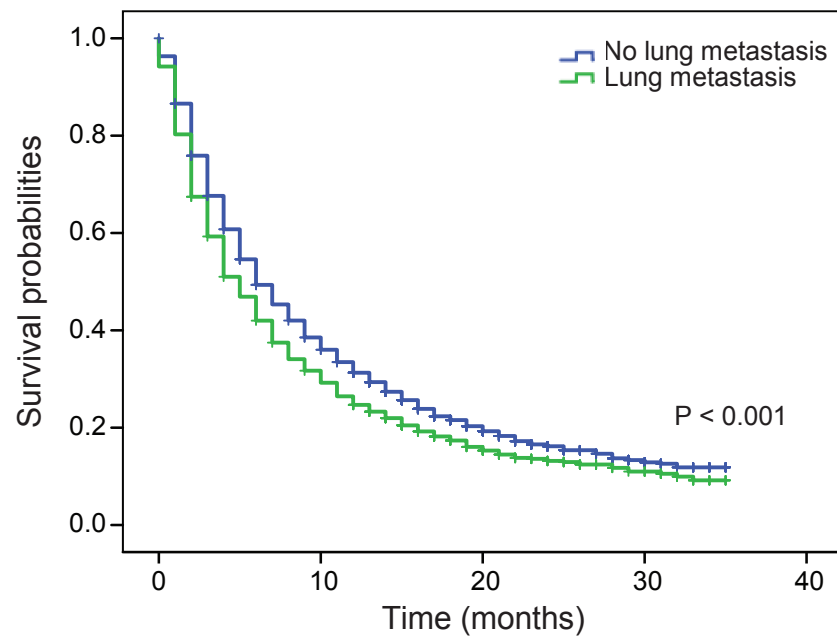

D

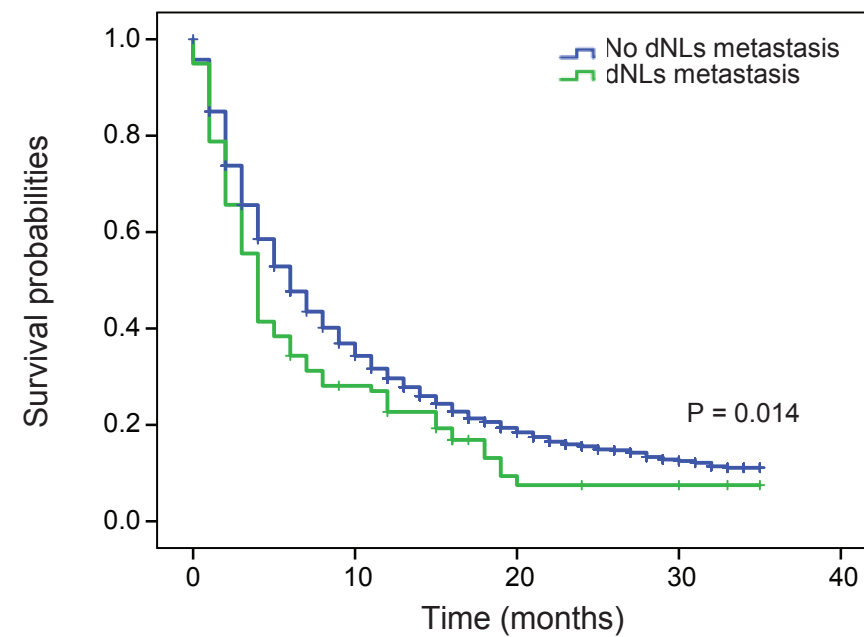

Supplementary figure 6

Supplement: Supplementary file 6 — Fig S6 [file CAM4-10-471-s006.pdf]

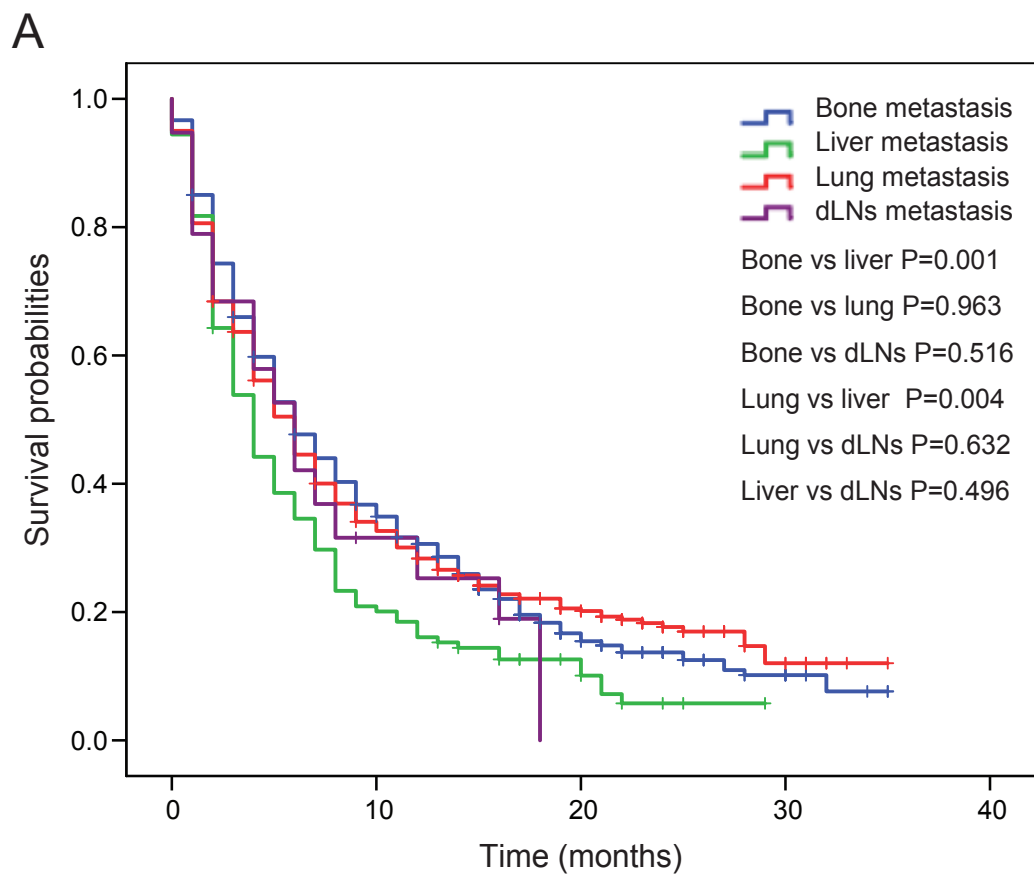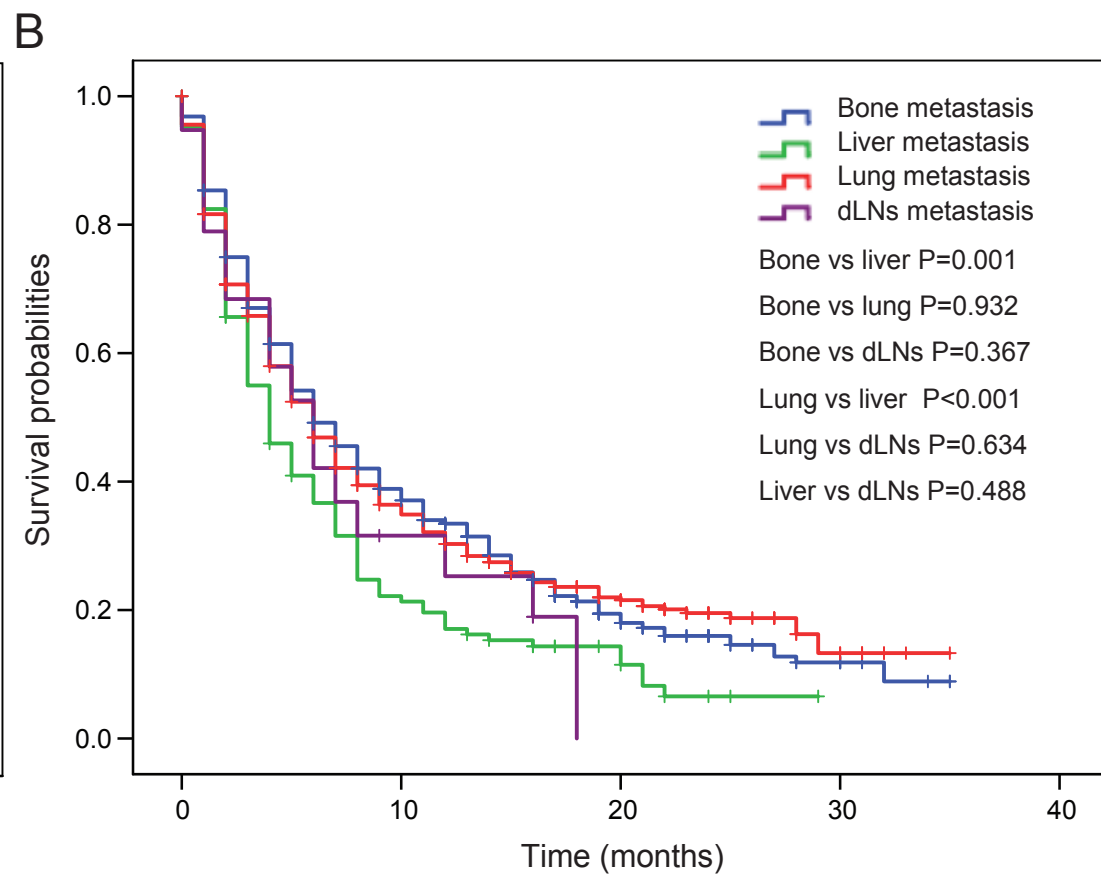

Supplementary figure 7

Supplement: Supplementary file 7 — Fig S7 [file CAM4-10-471-s007.pdf]
