# Supplementary material for: Prognostic impacts of extracranial metastasis on non‐small cell lung cancer with brain metastasis: A retrospective study based on surveillance, epidemiology, and end results database
Source: Cancer Med. 2020 Dec 15;10(2):471–82. doi: 10.1002/cam4.3562 (PMC7877345; doi:10.1002/cam4.3562)
Supplement: Supplementary file 4 — Fig S4 [file CAM4-10-471-s004.pdf]

A

Variables	OS		
	HR	95%CI	P value
Age (21-49 vs 50-59 vs >=60y)	1.25	(1.18,1.33)	<0.001
Race(white vs black vs other vs unknow)	0.85	(0.80,0.90)	<0.001
Gender (famale vs male)	1.18	(1.10,1.27)	<0.001
Marriage (Single vs married vs unknow*)	0.93	(0.87,0.99)	0.030
Grade (I vs II vs III vs IV vs unknow)	0.99	(0.96,1.01)	0.312
Pathology (AD vs SCC vs LCC vs Other*)	1.14	(1.09,1.19)	<0.001
Stage T (T1 vs T2 vs T3 vsT4)	1.08	(1.05,1.12)	<0.001
Stage N (N1vs N2 vsN3 vsN4 vs Nx)	1.07	(1.04,1.11)	<0.001
Primary site surgery (No vs Yes)	0.66	(0.45,0.98)	0.038
Radiotherapy (No vs Yes)	0.93	(0.75,1.14)	0.471
Chemotherapy (No vs Yes)	0.30	(0.27,0.32)	<0.001
Number of involved extracranial organs (0 vs 1 vs 2 vs >=3)	1.21	(1.16,1.26)	<0.001

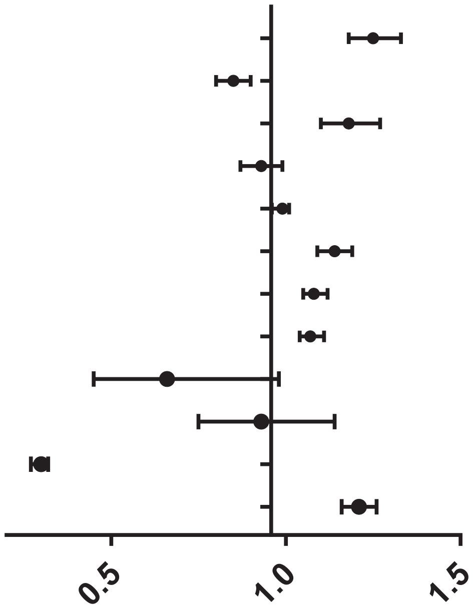

Abbreviation：HR：hazard ratio; 95% CI：95% confidence interval; AD: adenocarcinoma; SCC: squamous cell carcinoma; LCC: large cell carcinoma; OS: overall survival

B

Variables	CSS		
	HR	95%CI	P value
Age (21-49 vs 50-59 vs >=60y)	1.14	(1.07,1.21)	<0.001
Race(white vs black vs other vs unknow)	0.94	(0.88,0.99)	0.032
Gender (famale vs male)	1.04	(0.97,1.12)	0.290
Marriage (Single vs married vs unknow*)	0.98	(0.91,1.05)	0.482
Grade (I vs II vs III vs IV vs unknow)	1.02	(0.99,1.05)	0.327
Pathology (AD vs SCC vs LCC vs Other*)	1.09	(1.04,1.14)	<0.001
Stage T (T1 vs T2 vs T3 vsT4)	1.07	(1.03,1.11)	<0.001
Stage N (N1vs N2 vsN3 vsN4 vs Nx)	1.04	(1.00,1.08)	0.042
Primary site surgery (No vs Yes)	1.15	(0.78,1.71)	0.485
Radiotherapy (No vs Yes)	0.91	(0.73,1.13)	0.391
Chemotherapy (No vs Yes)	0.34	(0.31,0.37)	<0.001
Number of involved extracranial organs (0 vs 1 vs 2 vs >=3)	1.14	(1.10,1.19)	<0.001

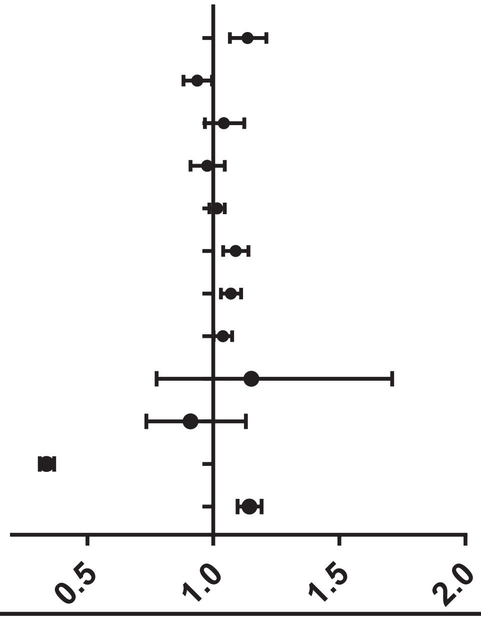

Abbreviation：HR：hazard ratio; 95% CI：95% confidence interval; AD: adenocarcinoma; SCC: squamous cell carcinoma; LCC: large cell carcinoma; CSS: cancer-specific survival

Supplementary figure 4
