# Supplementary material for: Prognostic impacts of extracranial metastasis on non‐small cell lung cancer with brain metastasis: A retrospective study based on surveillance, epidemiology, and end results database
Source: Cancer Med. 2020 Dec 15;10(2):471–82. doi: 10.1002/cam4.3562 (PMC7877345; doi:10.1002/cam4.3562)
Supplement: Supplementary file 8 — Table S1 [file CAM4-10-471-s008.docx]

**Supplementary Table 1 Characteristics of NSCLC patients with brain metastasis**

| **Characteristics** | **The number of involved extracranial organs** | | | | | **P value** |
| --- | --- | --- | --- | --- | --- | --- |
|  | **Total** | **0** | **1** | **2** | **≥3** |  |
|  | 3561 (100%) | 1588 (44.59%) | 1107 (31.09%) | 647 (18.07%) | 219(6.15%) |  |
| **Histopathology** |  | | | | | 0.255 |
| Adenocarcinoma | 2784 (78.18%) | 1214(76.45%) | 875(79.04%) | 512(79.13%) | 183(83.56%) |  |
| Squamous cell carcinoma | 478 (13.42%) | 229(14.42%) | 141(12.74%) | 86(13.29%) | 22(10.05%) |  |
| Large cell carcinoma | 101 (2.84%) | 55(3.46%) | 31(2.80%) | 12(1.85%) | 3(1.37%) |  |
| Other | 198 (5.56%) | 90(5.67%) | 60(5.42%) | 37(5.72%) | 11(5.02%) |  |
| **Age** |  | | | | | **0.022** |
| 21-49 | 262 (7.36%) | 98(6.17%) | 81 (7.32%) | 56(8.66%) | 27 (12.33%) |  |
| 50-59 | 962 (27.01%) | 433 (27.27%) | 287 (25.93%) | 181(27.98%) | 61 (27.85%) |  |
| **≥**60 | 2337 (65.63%) | 1057 (66.56%) | 739 (66.76%) | 410(63.37%) | 131 (59.82%) |  |
| **Race** |  | | | | | **<0.001** |
| White | 2746 (77.11%) | 1241 (78.15%) | 858(77.51%) | 476(73.57%) | 171(78.08%) |  |
| Black | 445 (12.50%) | 226(14.23%) | 124(11.20%) | 75(11.59%) | 20(9.13%) |  |
| Other^#^ | 370 (10.39%) | 121(7.62%) | 125(11.29%) | 96(14.84%) | 28(12.79%) |  |
| **Gender** |  | | | | | 0.067 |
| Male | 1768 (49.65%) | 824(51.89%) | 517(46.70%) | 321(49.61%) | 106(48.40%) |  |
| Female | 1793 (50.35%) | 764(48.11%) | 590(53.30%) | 326(50.39%) | 113(51.60%) |  |
| **Marital status** |  | | | | | 0.054 |
| Married | 1550(43.53%) | 726 (45.72%) | 453(40.92%) | 279(43.12%) | 92 (42.01%) |  |
| Unmarried | 1918 (53.86%) | 811(51.07%) | 628(56.73%) | 356 (55.02%) | 123 (56.16%) |  |
| Unknown | 93(2.61%) | 51(3.21%) | 26 (2.35%) | 12 (1.85%) | 4 (1.83%) |  |
| **Grade** |  | | | | | **<0.001** |
| Well | 75(2.11%) | 27(1.70%) | 29 (2.62%) | 15(2.32%) | 4 (1.83%) |  |
| Moderately | 462 (12.97%) | 225(14.17%) | 127 (11.47%) | 88 (13.60%) | 22(10.05%) |  |
| Poorly | 963 (27.04%) | 481(30.29%) | 293(26.47%) | 139 (21.48%) | 50 (22.83%) |  |
| Undifferentiated | 39 (1.10%) | 23 (1.45%) | 11 (0.99%) | 5 (0.77%) | 0 (0.00%) |  |
| Unknown | 2022 (56.78%) | 832 (52.39%) | 647(58.45%) | 400 (61.82%) | 143 (65.30%) |  |
| **Stage T** |  | | | | | **<0.001** |
| T1 | 456 (12.81%) | 271 (17.07%) | 131 (11.83%) | 47(7.26%) | 7(3.20%) |  |
| T2 | 1019 (28.62%) | 574 (36.15%) | 304 (27.46%.) | 122(18.86%) | 19(8.68%) |  |
| T3 | 935 (26.26%) | 383 (24.12%) | 281 (25.38%) | 202(31.22%) | 69(31.51%) |  |
| T4 | 1511 (32.32%) | 360 (22.67%) | 391 (35.32%) | 276(42.66%) | 124(56.62%) |  |
| **Stage N** |  | | | | | **<0.001** |
| N0 | 769(21.60%) | 449(28.27%) | 212(19.15%) | 89(13.76%) | 19(8.68%) |  |
| N1 | 302(8.48%) | 156(9.82%) | 88 (7.95%) | 43(6.65%) | 15(6.85%) |  |
| N2 | 1582(44.43%) | 689(43.39%) | 492 (44.44%) | 308(47.60%) | 93(42.47%) |  |
| N3 | 828(23.25%) | 261(16.44%) | 289 (26.11%) | 194(29.98%) | 84(38.36%) |  |
| NX | 80(2.25%) | 33(2.08%) | 26 (2.35%) | 13(2.01%) | 8(3.65%) |  |
| **Surgery** |  | | | | | 0.285 |
| Yes | 41(1.15%) | 24(1.51%) | 11 (0.99%) | 5(0.77%) | 1 (0.46%) |  |
| No | 3520(98.85%) | 1564 (98.49%) | 1096 (99.01%) | 642(99.23%) | 218 (99.54%) |  |
| **Radiation** |  | | | | | 0.651 |
| Yes | 3456(97.05%) | 1543 (97.17%) | 1077 (97.29%) | 623(96.29%) | 213 (97.26%) |  |
| No | 105(2.95%) | 45(2.83%) | 30 (2.71%) | 24(3.71%) | 6 (2.74%) |  |
| **Chemotherapy** |  | | | | | 0.578 |
| Yes | 2390(67.12%) | 1061 (66.81%) | 748 (67.57%) | 426(65.84%) | 155 (70.78%) |  |
| No | 1197(32.88%) | 527 (33.19%) | 359 (32.43%) | 221(34.16%) | 64 (29.22%) |  |

Abbreviation: NSCLC: non-small cell lung cancer; ^#^: Other races included American Indians, AK Natives, Asians and Pacific Islanders
