# Supplementary material for: Prognostic impacts of extracranial metastasis on non‐small cell lung cancer with brain metastasis: A retrospective study based on surveillance, epidemiology, and end results database
Source: Cancer Med. 2020 Dec 15;10(2):471–82. doi: 10.1002/cam4.3562 (PMC7877345; doi:10.1002/cam4.3562)
Supplement: Supplementary file 9 — Table S2 [file CAM4-10-471-s009.docx]

**Supplementary table 2 Survival analysis among different numbers of involved extracranial organs**

| **Survival rate** | **The number of involved extracranial organs** | | | | **P value** |
| --- | --- | --- | --- | --- | --- |
|  | **0** | **1** | **2** | **≥3** |  |
| **6-month OS** | **52.9%** | **45.1%** | **40.6%** | **37.9%** | **<0.001** |
| **1-year OS** | **34.0%** | **28.1%** | **24.4%** | **18.5%** | **<0.001** |
| **6-month CSS** | **39.3%** | **33.4%** | **30.9%** | **32.0%** | **<0.001** |
| **1-year CSS** | **15.6%** | **13.7%** | **12.3%** | **10.3%** | **<0.001** |
