# Supplementary material for: Prognostic impacts of extracranial metastasis on non‐small cell lung cancer with brain metastasis: A retrospective study based on surveillance, epidemiology, and end results database
Source: Cancer Med. 2020 Dec 15;10(2):471–82. doi: 10.1002/cam4.3562 (PMC7877345; doi:10.1002/cam4.3562)
Supplement: Supplementary file 10 — Table S3 [file CAM4-10-471-s010.docx]

**Table 3 Univariate analyses of OS and CSS in diverse extracranial metastatic organs**

| **Variable** | **OS** |  | **CSS** |  |
| --- | --- | --- | --- | --- |
|  | **HR (95% CI)** | **P value** | **HR (95% CI)** | **P value** |
| **Bone (metastasis vs no metastasis)** | **1.20(1.12-1.29)** | **<0.001** | **1.10 (1.02-1.18)** | **0.017** |
| **Liver (metastasis vs no metastasis)** | **1.42(1.30-1.56)** | **<0.001** | **1.23(1.12-1.36)** | **<0.001** |
| **Lung (metastasis vs no metastasis)** | **1.19(1.10-1.29)** | **<0.001** | **1.12(1.03-1.22)** | **0.007** |
| **dLNs (metastasis vs no metastasis)** | **1.29(1.04-1.60)** | **0.020** | **1.20(0.96-1.50)** | **0.104** |

Abbreviation: OS: overall survival; CSS: cancer-specific survival; dLNs: distant lymph nodes; HR：hazard ratio; 95% CI：95% confidence interval
